# Pembrolizumab as a first line therapy in a patient with extensive mucoepidermoid salivary gland carcinoma. A complete clinical, radiological and pathological response. A very specific case

**DOI:** 10.1007/s12672-022-00502-4

**Published:** 2022-05-28

**Authors:** Raed Farhat, Noam Asna, Yaniv Avraham, Ashraf Khater, Majd Asakla, Alaa Safia, Sergio Szvalb, Nidal Elkhatib, Shlomo Merchavy

**Affiliations:** 1 Otolaryngology, Head & Neck Surgery Unit, Ziv Medical Center, Golan Heights, 1028 Safed, Israel; 2 Oncology Institute, Ziv Medical Center, Zefat, Israel; 3 Pathology Institute, Ziv Medical Center, Zefat, Israel

**Keywords:** Pembrolizumab, Salivary gland cancers (SGCs): immunotherapy, Complete response (CR)

## Abstract

**Background:**

Patients with advanced salivary gland malignancies (SGCs) have few therapy options. Although results from newly published trials suggest that checkpoint inhibition may be useful in a subgroup of patients, there are no clear criteria for PD-L1 score in SGCs. Chemotherapy benefits were observed to be limited, with a dismal prognosis in unresectable and high-grade SGC. Immunotherapies have demonstrated extraordinary efficacy in a variety of cancers, including non-small cell lung cancer and malignant melanoma. Anti-PD-1 antibody pembrolizumab has been shown to have potent anti-tumor action in a number of clinical trials.

**Case presentation:**

We report a unique case of advanced high grade mucoepidermoid carcinoma of the parotid salivary gland after Pembrolizumab treatment as a first line therapy. The tumor was downstaged as a result of the pembrolizumab treatment, allowing for a successful surgical excision with no facial nerve sacrifice and no major neoadjuvant treatment adverse effects, and the final specimen pathology was tumor-free. In these types of malignancies, a similar technique resulted in a complete response (CR) radiologically and pathologically has never been discussed before.

**Conclusions:**

In pretreated patients with high-grade salivary gland mucoepidermoid carcinoma, pembrolizumab showed good anticancer activity and provided a clinically, radiologically, and pathological response with a viable treatment choice. More research is needed to bring Pembrolizumab to the front-line of treatment. The time and duration of medication should be compared to the time required for surgery in these investigations.

## Introduction

Salivary gland carcinoma (SGC) is a rare disease that accounts for just 6% of all head and neck cancers [1]. Surgical excision with or without postoperative adjuvant radiation therapy is the curative treatment for most histological subtypes [2–4].

Mucoepidermoid carcinoma (MEC) is a malignant tumor that primarily arises from the salivary glands; In minor salivary gland malignancies, MEC most commonly is found [5]. In both children and adults, MEC is the most common malignant salivary gland tumor [6].

In extremely aggressive subtypes such salivary duct carcinomas, adjuvant treatment options such as chemotherapy or radiation had no discernible effect on survival [7]. Patients with unresectable primaries/recurrences or patients with distant metastasis [2] have no standard treatment or demonstrated efficacy. Chemotherapies such as cisplatin, doxorubicin, and cyclophosphamide have been documented to have modest benefit, with a poor prognosis [2, 8–10].

Immunotherapies have demonstrated extraordinary efficacy in a variety of cancers, including non-small cell lung cancer and malignant melanoma. All of these treatments work in the same way, by guiding the body's own immune system to eliminate tumor cells [11, 12].

Pembrolizumab is a monoclonal antibody this is absolutely humanized immunoglobulin G4/anti-PD-1. Pembrolizumab has proven sturdy antitumor pastime and a positive protection profile in a lot of tumor types, and it's far presently authorized in extra than 60 nations for 1 or extra superior malignancies, which includes recurrent or metastatic head and neck squamous carcinoma that advanced on or after platinum-primarily based totally chemotherapy withinside the United States [13–15].

With the inclusion of immunotherapy, either alone or in combination with chemotherapy, the newly released KEYNOTE-048 trial established a new standard in this situation [16].

This article reports a unique case of unresectable advanced high grade mucoepidermoid carcinoma of the salivary gland with complete response after Pembrolizumab treatment as a first line therapy. A similar strategy has never been tried in high grade salivary gland tumors, and complete radiological and pathological responses have never been reported before in this kind of tumors.

This treatment resulted in downstaging of the tumor and led to its successful surgical resection.

## Case presentation

A 68-year-old male, diabetic with a forty pack-year smoking history presented with an enlarging left, slightly tender, rapidly progressive swelling of the left parotid gland during the previous 6 months before our first examination. The patient was referred to head and neck clinic, Department of Otolaryngology Head and Neck Surgery, Ziv Medical Center in Safed, in the north of Israel.

**Fig. 1:**
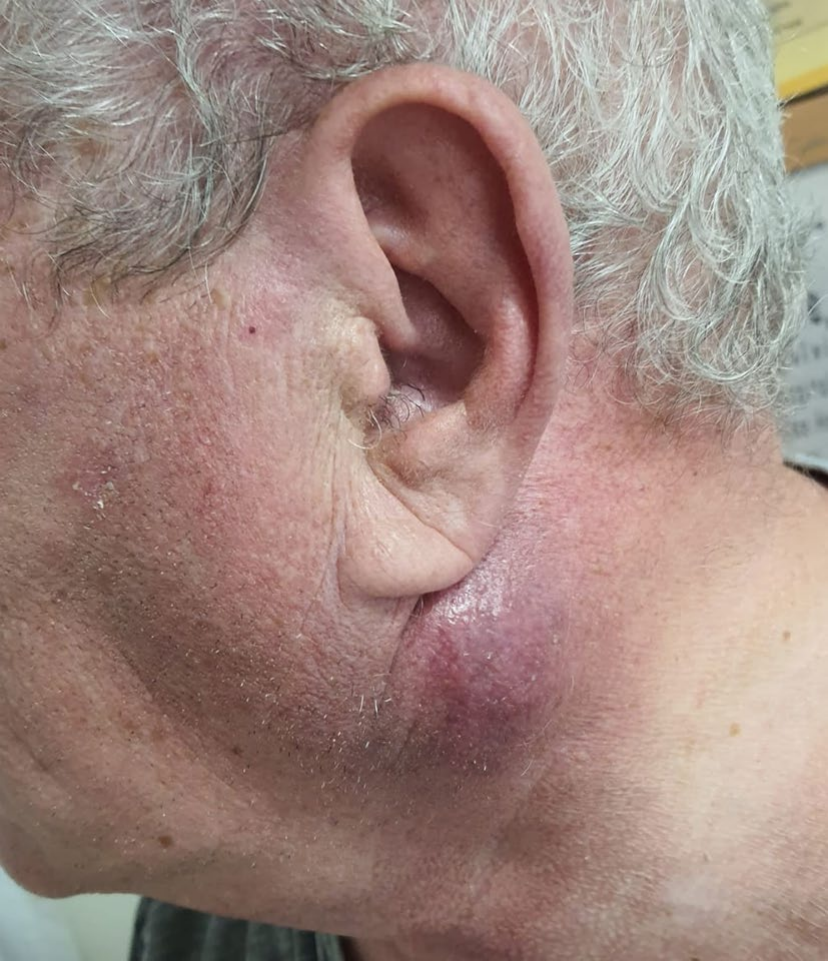
Firm, immobile, slightly tender, 3*3 cm left parotid mass with overlying skin changes

Physical examination showed a firm, immobile, slightly tender, 3 × 3 cm left parotid mass with overlying skin change (Fig. 1), with multiple hard, immobile lymph nodes, 3×2 cm, at level II,III,V of the left neck, the remainder of the head and neck examination was unremarkable. Flexible nasendoscopy findings of the nasopharynx, oropharynx, and larynx were unremarkable.

**Fig. 2:**
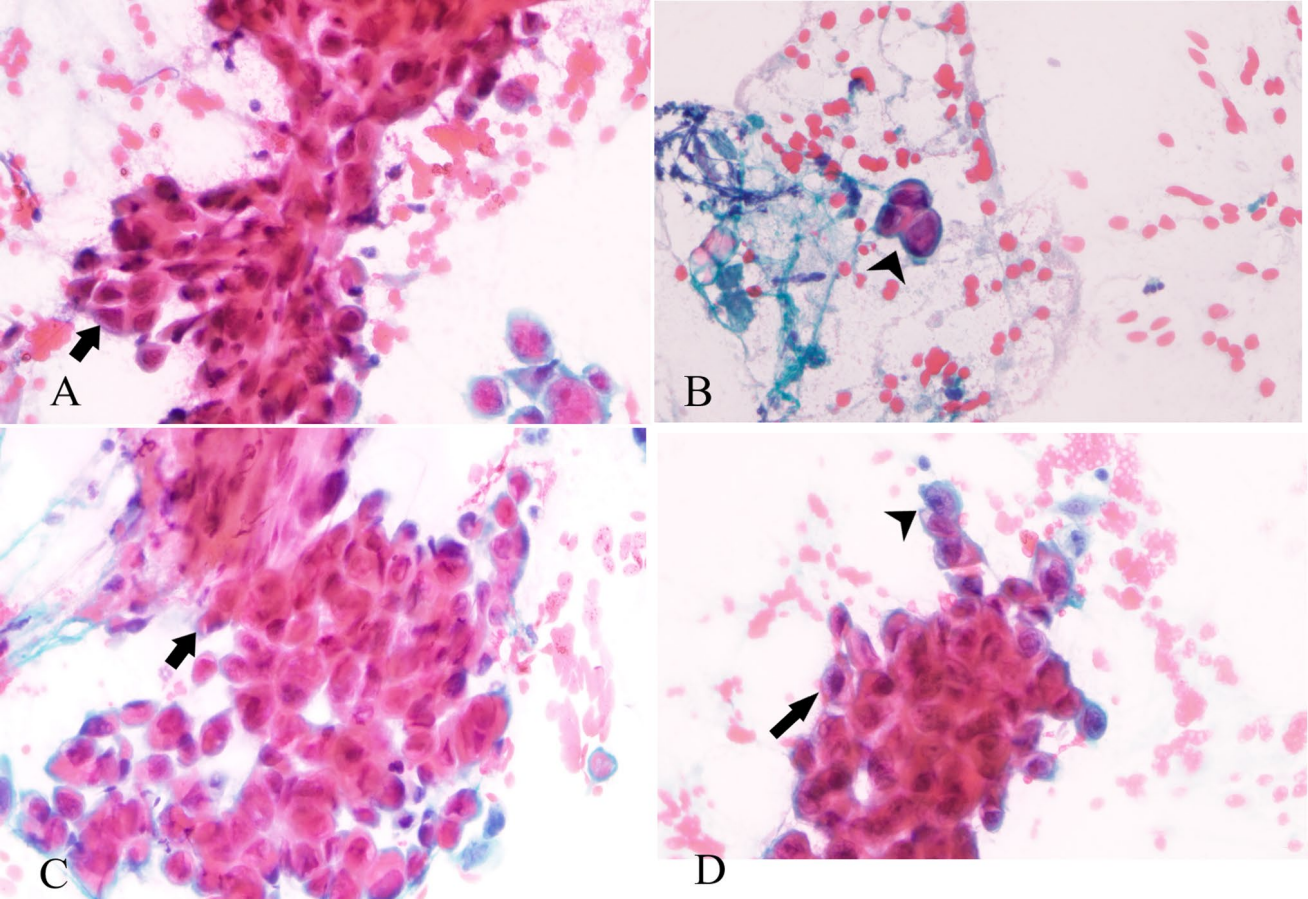
Microphotograph of fine needle aspiration cytology pathological findings that support the diagnosis of high grade MEC from the parotid gland origin (X40 H&E). **A**, **C** Smears groups of atypical epithelial cells (black arrow) with eosinophilic cytoplasm with enlarged and irregular nuclei, prominent nucleoli. **B** Smear showing some mucin-secreting vacuolated cells (black arrowhead). **D** Some mucin-secreting (black arrowhead) intermixed with atypical epithelial cells (black arrow)

Fine needle aspiration (FNA) of the left parotid mass was consistent with positive malignant cells for high grade mucoepidermoid carcinoma with mitosis (Fig. 2).

**Fig. 3:**
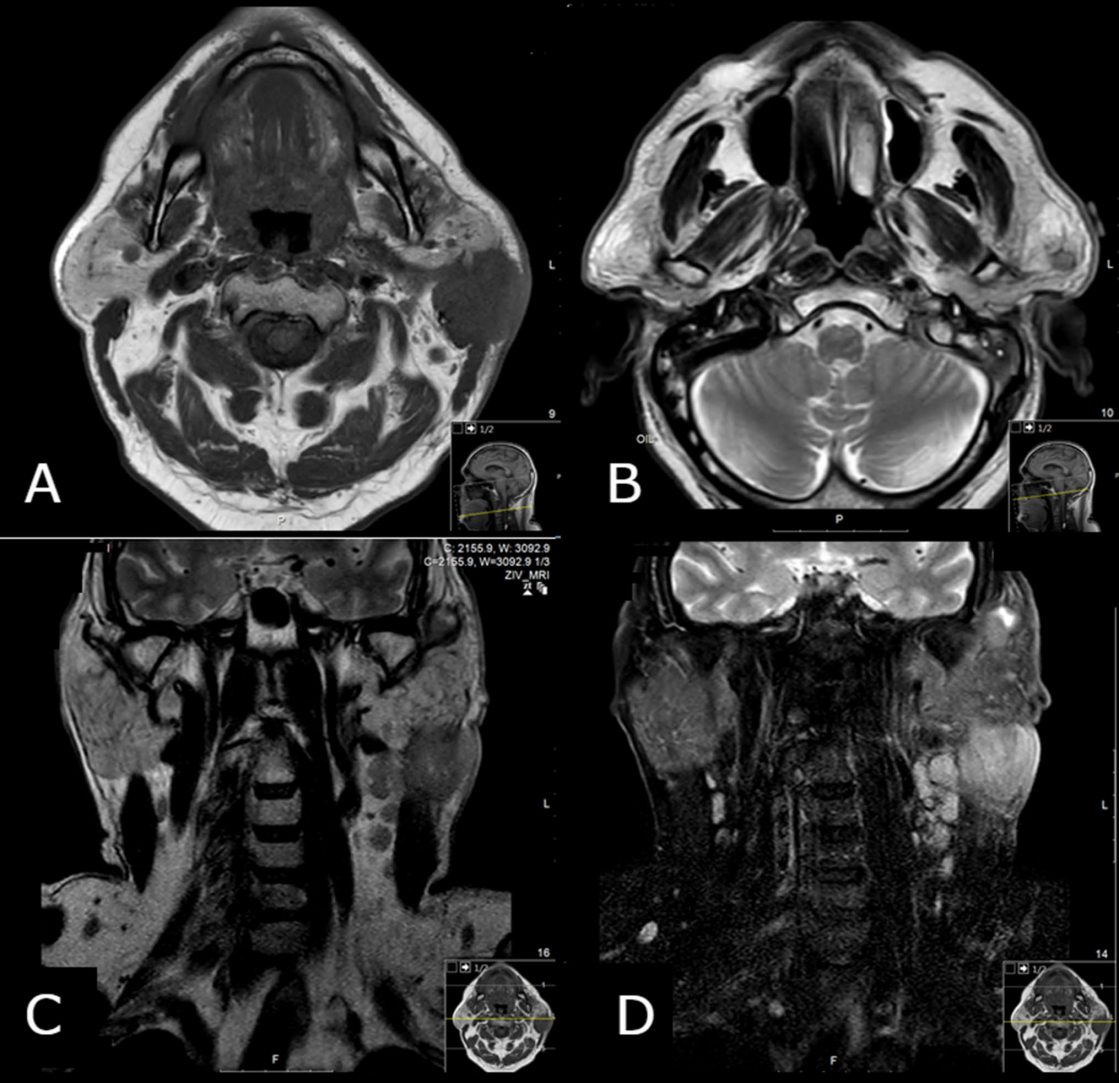
**A** MRI T1 lobulated, irregular mass, located in the posteroinferior segment of the superficial segment of the superficial lobe in the left parotid gland. **B** MRI T2 with areas of extensive central necrosis, septation. **C** Several suspected lymph nodule in the left infraparotid area and level II and III. **D** Peripheral wall enhancement

Contrast-enhanced magnetic resonance imaging (MRI) revealed a lobulated, irregular mass, 37*39*47 mm located in the posteroinferior segment of the superficial and deep lobe in the left parotid gland, with areas of extensive central necrosis, septation, and peripheral wall enhancement contiguous with the anterior portion of external auditory canal and SCM muscle with subcutaneous tissue and skin involvement, several nodules in the left infraparotid area and level II were calcified (Fig. 3).

**Fig. 4:**
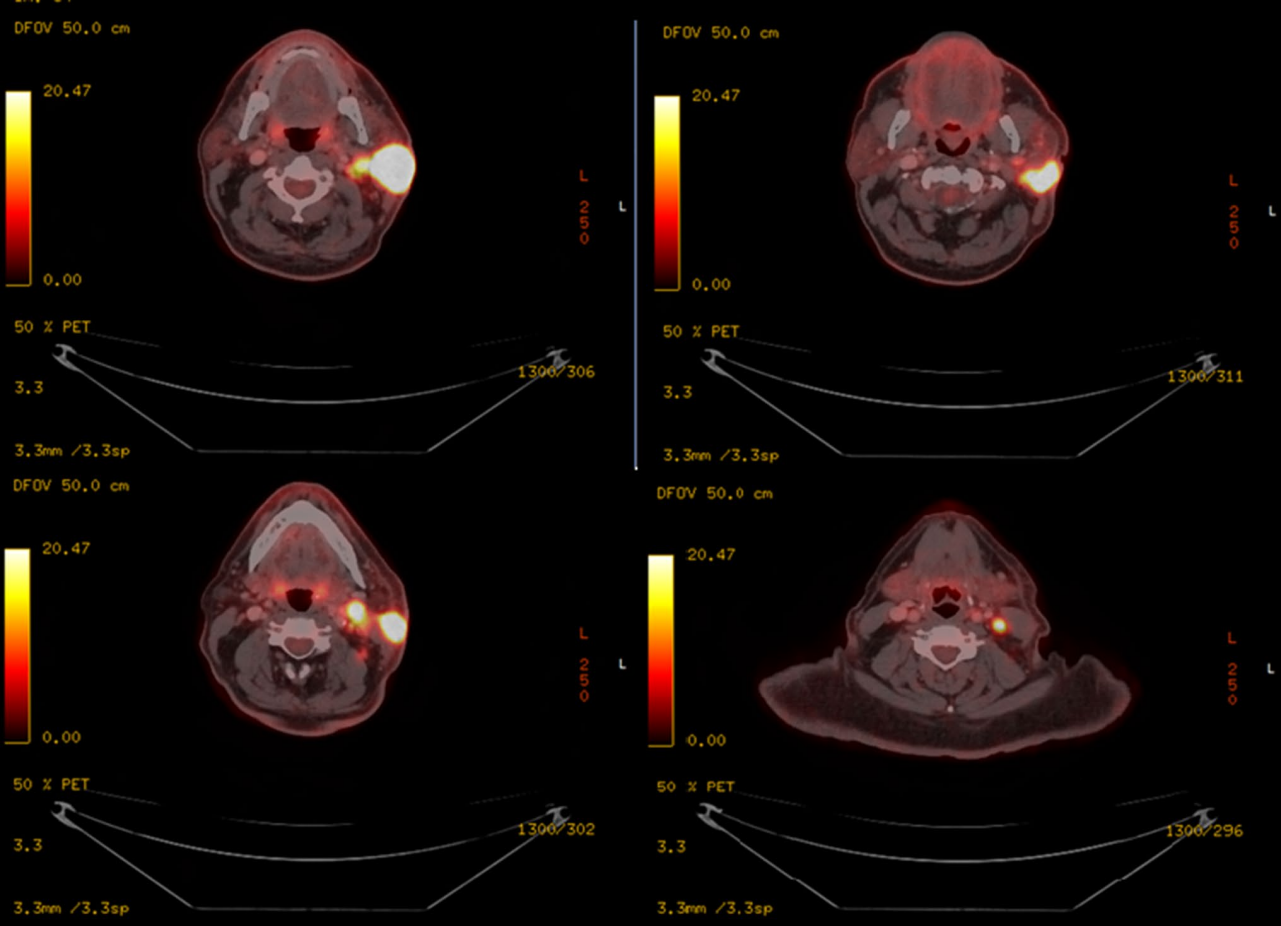
. The patient with metabolically active tumor prior to the initiation of immunotherapy with Pembrolizumab

The 18F-FDG PET/CT revealed hypermetabolic activity within the mass in the left parotid gland with involvement of the skin, SCM muscle and several nodules in the left infraparotid area and level II,III in the left neck (Fig. 4).

The final diagnosis was clinical stage IV (T4aN2bM0).

The patient was considered as high-grade MECs which progressed rapidly and caused pain, soft tissue invasion; these MECs are associated with poor overall survival, which approaches 40% to 50% at 5 years, and with an increased risk for locoregional and distant failures [17–19].

The recommended therapy for high-grade MECs includes surgical resection with selective neck dissection followed by adjuvant radiotherapy [20].

Pembrolizumab, as a single agent, is indicated for the first-line treatment of patients with metastatic or with unresectable, recurrent HNSCC, FDA-approved [21].

SCC and high grade MEC have similar histopathologic features and presence of intermediate and mucus cells are the only key for differentiation [22, 23].

In our case, the high morbidity and mortality associated with the need of extensive surgical resection with free flap reconstruction and the massive loss of tissue with possible facial nerve sacrifice, in addition to increased risk for locoregional and distant failures led us to think about neoadjuvant treatment.

Pembrolizumab monotherapy was generally well tolerated in advanced Salivary Gland Carcinoma (SGC), with a safety profile that reflects previous experience of pembrolizumab in patients with advanced cancers [24].

Treatment plan was made by a multidisciplinary team and after multiple discussions with the goal of maximizing survival with preservation of form and function.

Our patient received pembrolizumab intravenously at 200 mg every 2 weeks, with a good compliance.

From September 2021 to November 2021, he underwent 2 cycles of pembrolizumab, the drug was well tolerated with minimal side effects, nausea and fatigue.

**Fig. 5:**
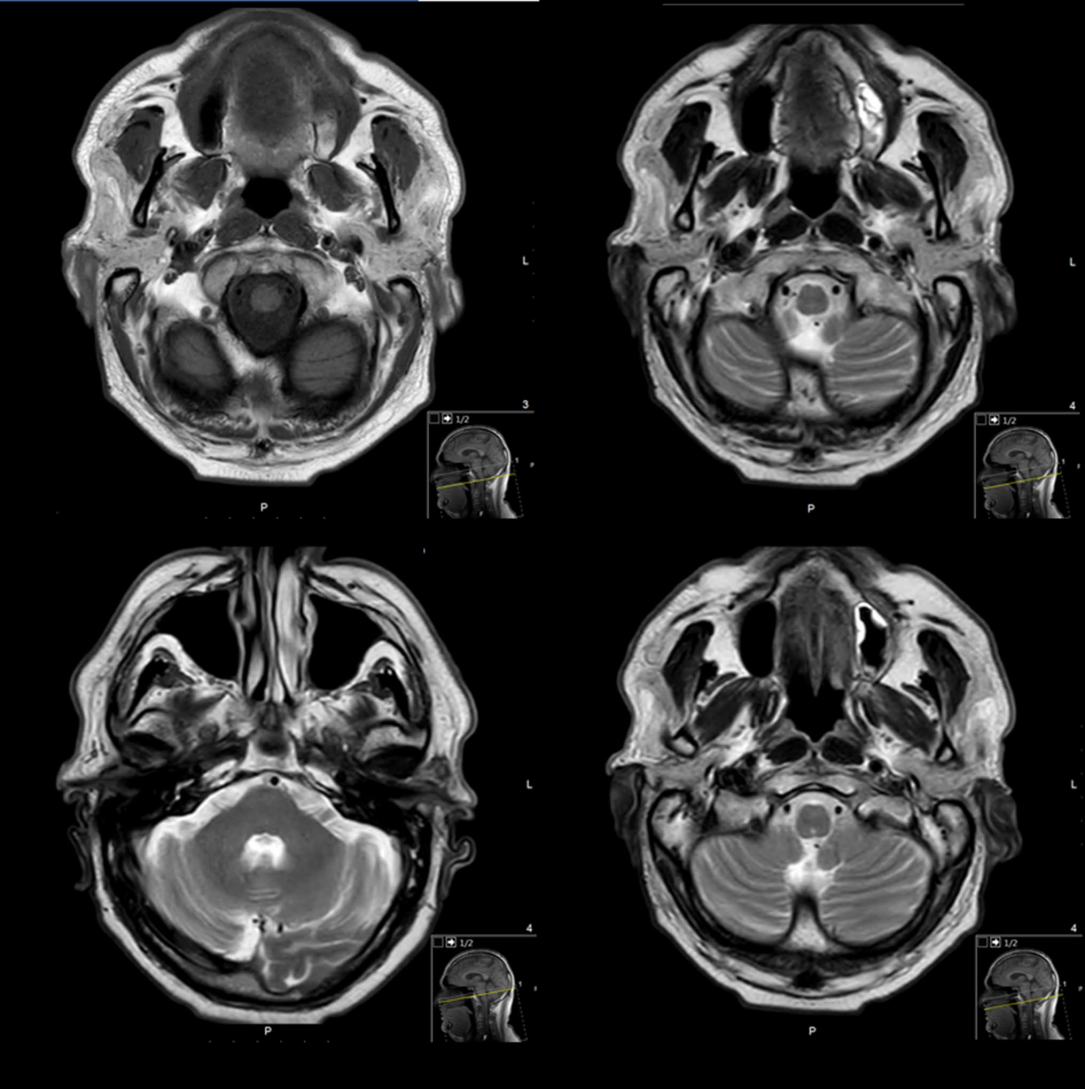
MRI T1 and T2—after 2 cycles of the neoadjuvant treatment (after 8 weeks) shows significant decrease in the tumor or even its disappearance, attained a complete remission

**Fig. 6:**
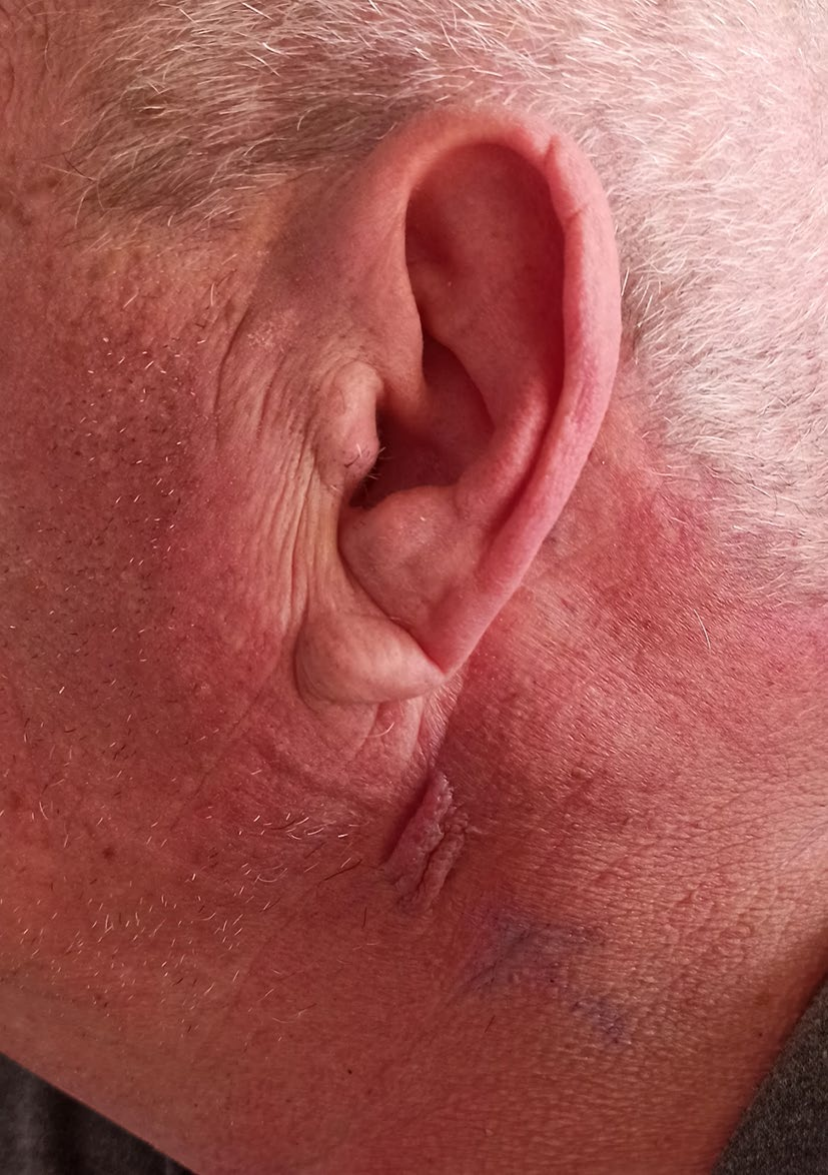
. The mass appearance after 2 cycles of neoadjuvant treatment (after 8 weeks)

The MRI scan after 3 cycles, 8 weeks after the treatment initiation revealed a significant decrease in the tumor or even its disappearance and on follow-up, the patient had attained a complete remission in the clinic examination (Figs. 5, 6).

**Fig. 7:**
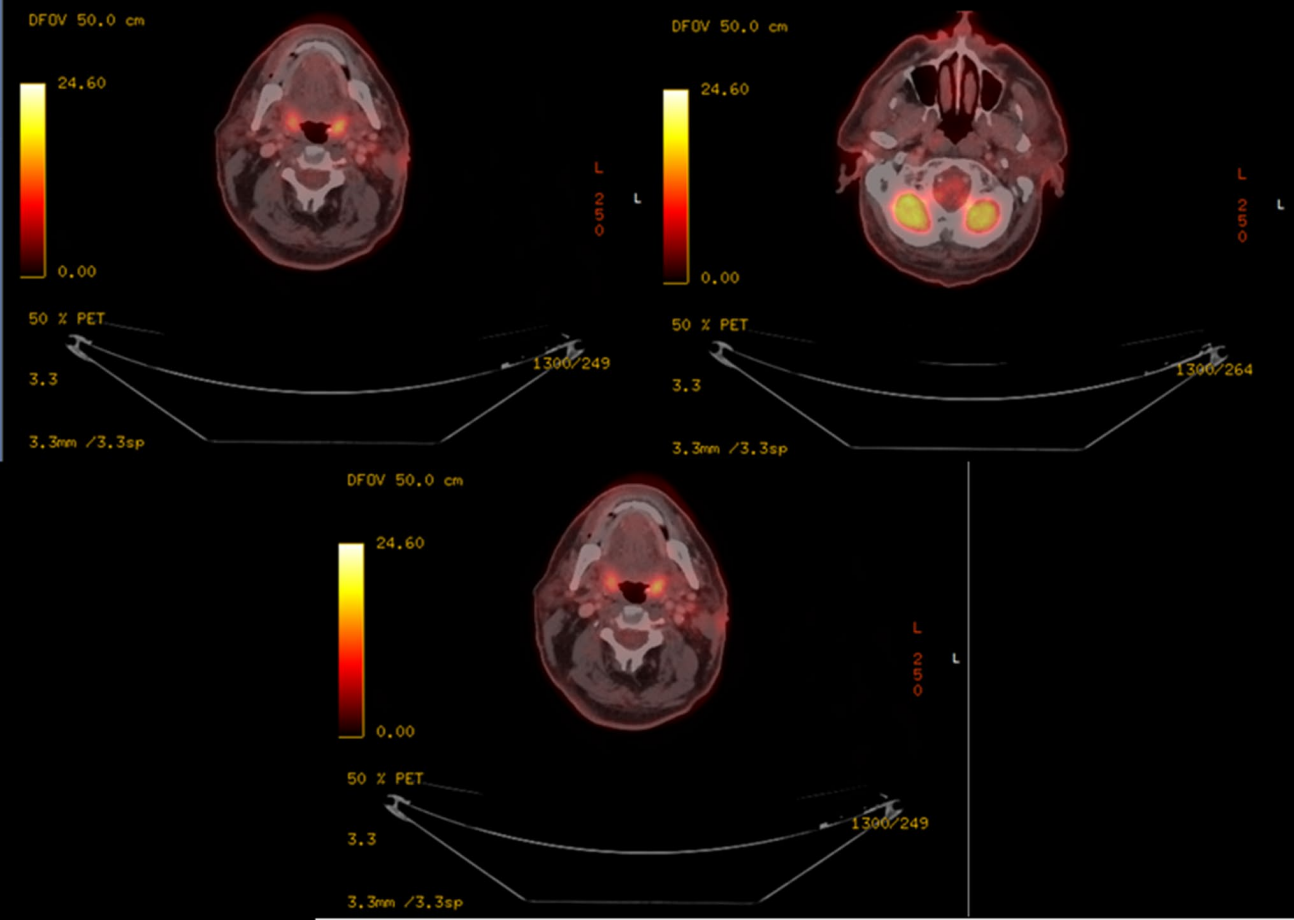
A complete metabolic response already 14 weeks after immunotherapy of Pembrolizumab initiation despite remaining morphological masses on CT

Restaging with 18FDG PET-CT at 6 weeks after completion of immunologic treatment, 14 weeks after the treatment initiation demonstrated a full resolution of the parotid gland metabolic activity. In the left neck, the nodal status had generally improved with no signs of hypermetabolic activity. High metabolic uptake was not seen elsewhere. (Fig. 7).

**Fig. 8:**
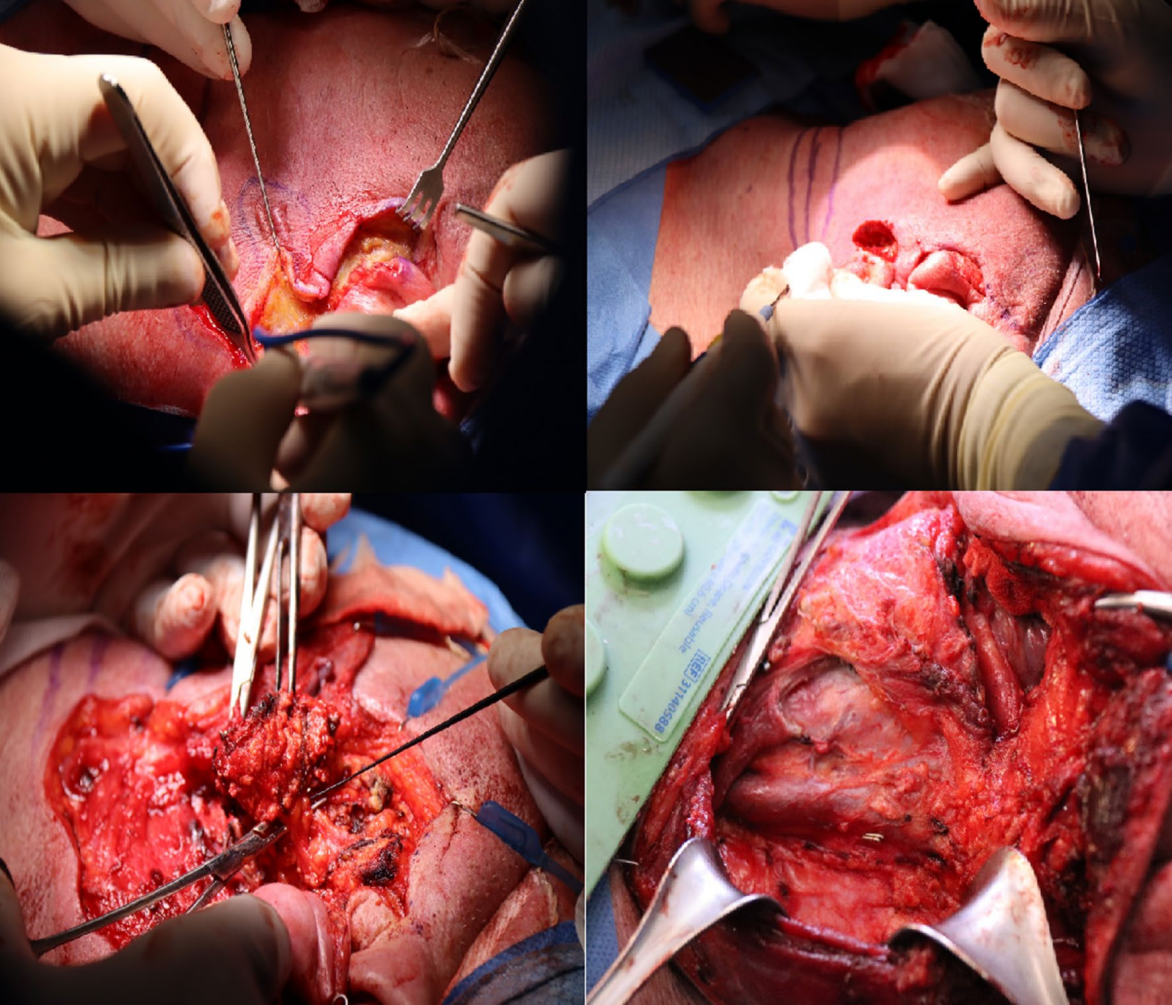
The patient underwent left parotidectomy with neck dissection and excision of the involoved skin

As a precaution, left parotidectomy and unilateral neck dissection levels I,II,III,IV were recommended for locoregional control and because of remaining morphological masses on ct. (Fig. 8).

**Fig. 9:**
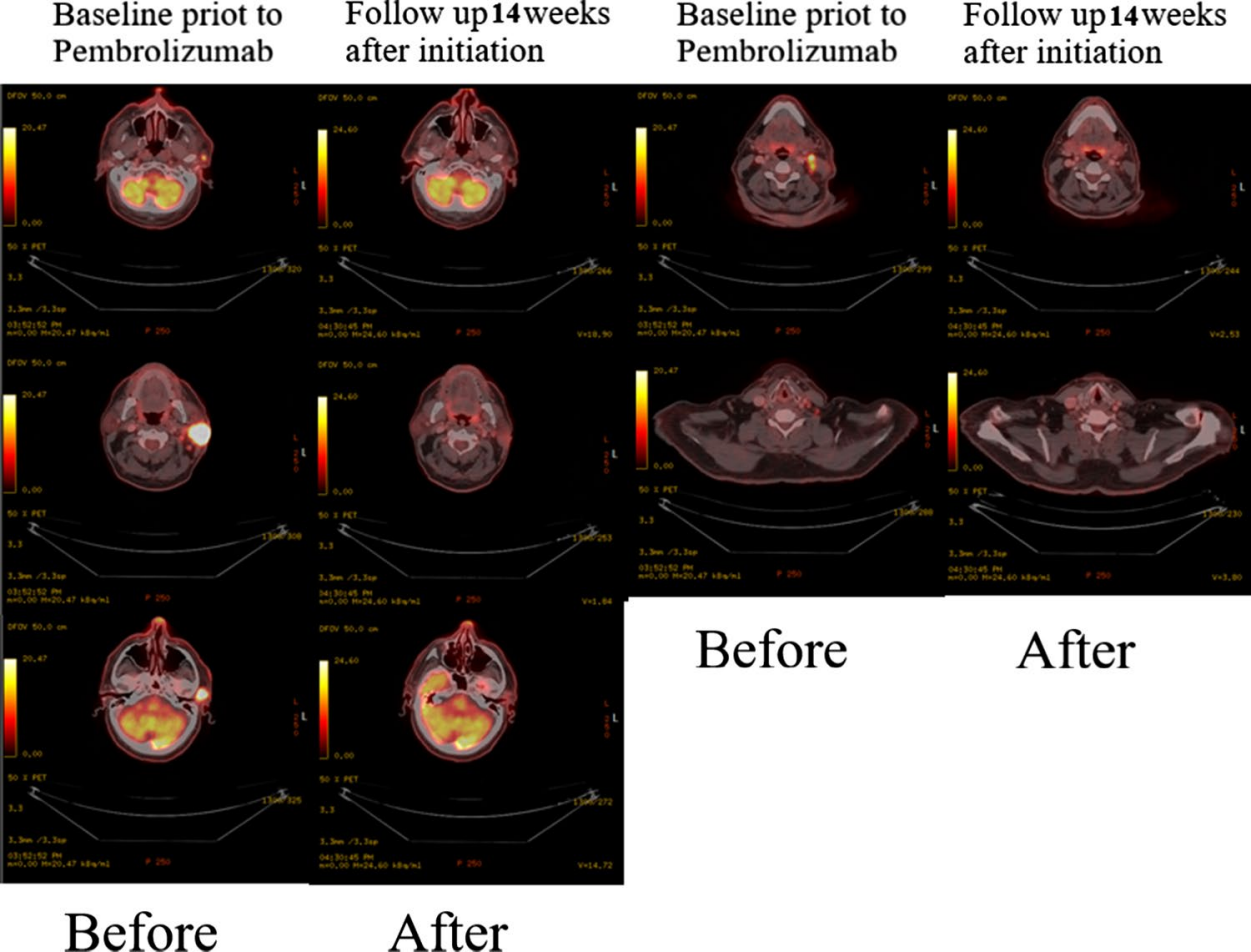
PET CT before and after treatment. Left side column—baseline prior to Pembrolizumab. Right side column—follow up 14 weeks after initiation

All the 18FDG PET-CT scans performed are summarized in (Fig. 9).

Final pathological results showed no evidence of carcinoma neither in the parotid gland nor in the neck lymph nodes and had free margins.

The patient has continued to be supervised with CT scans and serum tumor marker measurements every 3 or 4 months and is still in complete remission at this time.

## Discussion

This is the first report that describes complete resolution of high grade malignant salivary gland mucoepidermoid carcinoma with Pembrolizumab as a “first line” therapy treatment.

Cohen et al. [24] published the results of the phase Ib KEYNOTE-28 study, which used a single-agent pembrolizumab 10 mg/kg once every two weeks in 38 PDL1-expressing recurrent-metastatic salivary gland carcinomas. The majority of the included subjects had adenocarcinomas, and there was no evidence of progression prior to participation in this trial. Three of the 38 individuals enrolled had a Partial Response (PR), with an overall response rate of 12% and a median response duration of three months. There were no replies indicating complete remission.

There is no gold standard for the treatment of advanced SGC, and present medicines are often ineffective. Small studies, many of which were completed before present analytical concepts (e.g., RECIST) [25], are available to support the use of classical cytotoxic chemotherapy.

Small phase I and II clinical trials, again with heterogeneous histologies and variances in design and eligibility for such treatment, make up the prospective experience with antiPD1 checkpoint inhibitors in salivary gland carcinomas.

Although pembrolizumab has a primary site agnostic approval from the US Food and Drug Administration for mismatch repair–deficient cancers, it is crucial to highlight that this was based on a nine-patient cohort of non-colorectal cancer patients with no salivary gland malignancies [26].

In SGCs, the role of tumor mutation burden (TMB) is unknown. The KEYNOTE-158 trial's TMB subgroup analysis resulted to the approval of pembrolizumab as an agnostic therapy for patients with TMB > 10 mut/Mb. Three patients with salivary histology and elevated TMB were treated, with one of them achieving a partial response [23].

Hanno et al. concluded in their paper that using established scoring criteria for PD-L1 expression (combined positivity score (CPS) and immune cell (IC) score, tumor proportion score TPS), they were able to identify which types of adenocarcinomas, not otherwise specified (AC, NOS) among malignant salivary gland tumors would benefit most from immune checkpoint inhibition [27].

Another study discovered PD-L1 expression in 51 percent of malignant salivary gland tumor tissues. The presence of PD-L1 antibodies on tumor cell membranes was associated to patient prognosis, stage, and recurrence or metastasis after surgery.On the other hand, PD-L1 immunodetection of tumor-infiltrating mononuclear cells (TIMCs) has been associated to post-surgery recurrence or metastasis, as well as patient prognosis [28].

Although the current data from salivary gland malignancies trials are limited, no recommendations have been made regarding antiPD1 as first line treatment. our result in using Pembrolizumab as a first line agent in advanced high grade mucoepidermoid carcinoma suggests that this treatment may be a promising cancer therapy.

Moreover, data points cannot be based on only one case, this result cannot be generalized and we avoid making a recommendation.

More studies are needed to assess Pembrolizumab's efficacy in front-line and maintenance settings, as well as trials of combinations with chemotherapy, radiation, or other immune checkpoints.

## Conclusion

In conclusion, Pembrolizumab has demonstrated a promising antitumor activity in pre-treated patients with high grade salivary gland mucoepidermoid carcinoma, and offered a clinically, radiological and pathological response with a meaningful therapeutic option.

Further studies that move the treatment of Pembrolizumab to front-line, maintenance settings and combinations with other treatment methods are necessary. These studies should include the time and duration of the pharmacotherapy in relation to the needed time of surgery. Side effects of the drugs should also be followed as well as patient compliance.

## Data Availability

All data generated or analysed during this study are included in this published article [and its supplementary information files].
